# On the Medical Properties of Lupulus

**Published:** 1816-12

**Authors:** Henry Ward


*For the London Medical and Physical Journal.*

### *On the Medical Properties of Lupulus;* by HENRY WARD, Esq.

THE Humulus lupulus was first introduced into the Pharmacopœia as a tonic, narcotic, and aromatic. The first and last of which properties it possesses in a considerable degree; but, as to the narcotic principle, I think there is but very little, if any; indeed, in my opinion, it possesses no such effect. Some old writers assert, that it possesses very considerable lithontripic qualities; and Ray supposes, that the inhabitants of London have not been so subject to calculous complaints since its introduction in the art of porter brewing. If we may credit the report of Lobb in this instance, a very hard urinary calculus was softened by a decoction of the hop; but this, as well as the narcotic principle, I am induced to think as erroneous. A pillow of hops was at one time a popular remedy. Dr. De Roches asserts, that, from his experiments, he found it produce sleep in syphilitic, pectoral, and rheumatic complaints. It has also been used in cancerous affections in the form of an ointment, which is reported by Mr. Freake to have produced ease in that dreadful malady, when other applications have failed.

As the aromatic principle of the hop resides in a volatile oil, in the process of decoction it is dissipated, and the bitter property, which is contained in a species of tannin, is preserved in a very considerable degree. The preparations of the hop are blackened by the addition of the sulphate of

 iron, iron. As a tonic and stomachic medicine, the hop is, perhaps, inferior to few, but I think the tincture is far preferable to the extract; as the former retains the aromatic oil, while in the latter it is dissipated during the evaporation, and the medicine therefore deprived of its most efficacious part.

Wishing to know the real property of the hop, I took f. ʒiſs of the tincture, (procured from Apothecaries' Hall,) which did not produce the least effect; the following night, I took f. ʒiij. without the least variation from the effect produced by the preceding dose. After a lapse of three nights, I took f. ℥i. without the least narcotic effects. In two nights after this, I took ℈j. of the Ex. Humuli, and the following night ℈ij. I also administered it in many instances, all of which tended plainly to convince me that no preparation of this plant contains the narcotic principle which it was thought to do.

*Sloane-street, London;*

*November* 8, 1816.

---

*For the London Medical and Physical Journal.*

